# Hierarchically Nanostructured Transition Metal Oxides for Lithium‐Ion Batteries

**DOI:** 10.1002/advs.201700592

**Published:** 2018-01-03

**Authors:** Mingbo Zheng, Hao Tang, Lulu Li, Qin Hu, Li Zhang, Huaiguo Xue, Huan Pang

**Affiliations:** ^1^ School of Chemistry and Chemical Engineering Institute for Innovative Materials and Energy Yangzhou University Yangzhou 225002 Jiangsu P. R. China

**Keywords:** hierarchical nanostructures, lithium‐ion batteries, transition metal oxides

## Abstract

Lithium‐ion batteries (LIBs) have been widely used in the field of portable electric devices because of their high energy density and long cycling life. To further improve the performance of LIBs, it is of great importance to develop new electrode materials. Various transition metal oxides (TMOs) have been extensively investigated as electrode materials for LIBs. According to the reaction mechanism, there are mainly two kinds of TMOs, one is based on conversion reaction and the other is based on intercalation/deintercalation reaction. Recently, hierarchically nanostructured TMOs have become a hot research area in the field of LIBs. Hierarchical architecture can provide numerous accessible electroactive sites for redox reactions, shorten the diffusion distance of Li‐ion during the reaction, and accommodate volume expansion during cycling. With rapid research progress in this field, a timely account of this advanced technology is highly necessary. Here, the research progress on the synthesis methods, morphological characteristics, and electrochemical performances of hierarchically nanostructured TMOs for LIBs is summarized and discussed. Some relevant prospects are also proposed.

## 1 Introduction

The shortage of fossil fuels and increasingly deteriorating environmental pollution have become a threat for humans as the global economy rapidly develops. Thus, green power sources should be developed to replace conventional fossil fuels.^1^, ^2^, ^3^, ^4^, ^5^, ^6^ Solar energy, wind energy, and tidal energy are good alternatives because of their renewability and low pollution. However, these sources are usually restricted by their intermittence and poor storage efficiency.^7^, ^8^ Electrochemical energy storage provides a feasible approach to store electric energy from these sources.^9^, ^10^, ^11^, ^12^, ^13^, ^14^, ^15^, ^16^ Among various electrochemical energy storage devices, lithium‐ion batteries (LIBs) have drawn more and more attention because of their high energy density, long cycling life, and environmental friendliness.^17^, ^18^, ^19^, ^20^, ^21^

**Figure 1:**
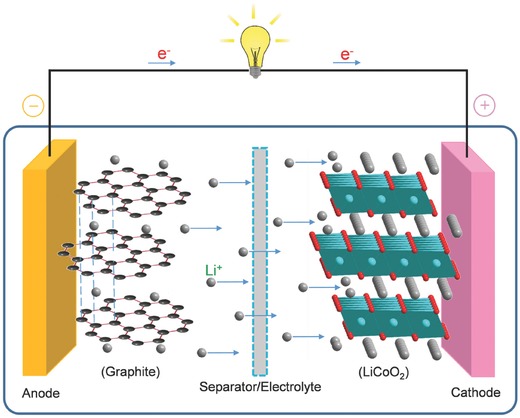
Schematic illustration of LIBs (LiCoO_2_/Li^+^ electrolyte/graphite).

**Figure 2:**
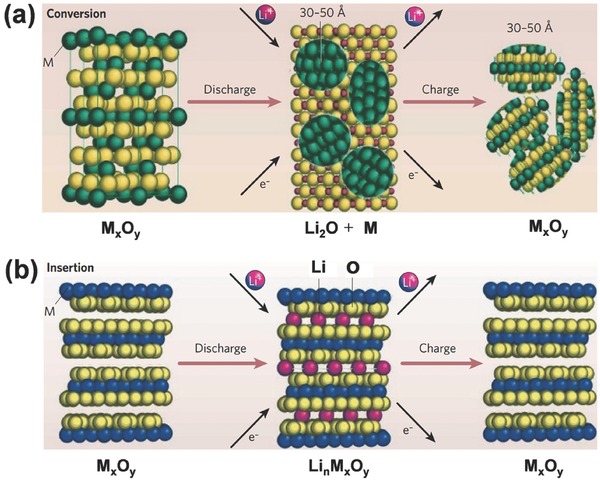
a) Schematic of the reaction mechanism based on conversion reaction. b) Schematic of the reaction mechanism based on intercalation/deintercalation reaction. Reproduced with permission.^36^ Copyright 2008, Nature Publishing Group.

LIBs typically consist of four main components: positive electrode, negative electrode, separator, and electrolyte (**Figure** **1**). The conversion between chemical energy and electrical energy can be achieved on the basis of the migration of Li ions across electrolytes between two electrodes and electron transmission through an external electrical circuit.^22^, ^23^ The performances of LIBs depend largely on the inherent properties of electrode materials.^24^ Transition metal oxides (TMOs) have become promising electrode materials for LIBs because of their multiple chemical valence states and diverse morphological characteristics.^25^, ^26^, ^27^, ^28^, ^29^ When used as anode materials, TMO‐based anodes have higher operating voltages in comparison to graphite‐based anodes. Metal lithium does not easily separate out on the surface of TMOs. Thus, TMOs possess better safety. In general, TMO electrode materials can be mainly classified into two types based on reaction mechanisms: (1) conversion reaction: M*_x_*O*_y_* + 2*y*Li^+^ + 2*y*e^−^ ↔ *y*Li_2_O + *x*M^30^, ^31^, ^32^, ^33^, ^34^ and (2) intercalation/deintercalation reaction: M*_x_*O*_y_* + *n*Li^+^ + *n*e^−^ ↔ Li*_n_*M*_x_*O*_y_*.^35^, ^36^, ^37^ Most of TMOs, such as iron oxides, cobalt oxides, nickel oxides, and manganese oxides, belong to the first type, and these TMOs usually possess high theoretical specific capacities. During cycling, M*_x_*O*_y_* is reduced to elemental metal (**Figure** **2**a). The main problem of TMOs with this mechanism is the serious volume variation during the cycling process and poor stability is consequently obtained. By contrast, other TMOs, such as titanium dioxide and vanadium pentoxide, are based on the intercalation/deintercalation mechanism and characterized by a relatively low theoretical specific capacity. In Figure 2b, this type of TMOs can host Li ions to generate Li*_n_*M*_x_*O*_y_* and maintain good structural integrity without collapsing during charge/discharge process and ensure a long cycle life.^38^ Therefore, the two types of TMOs possess own unique features when they are utilized as electrode materials for LIBs.

Excellent cycling stability and high rate performance are two vital factors for TMO electrode materials.^39^, ^40^ To achieve this goal, researchers focused on the design of rational and advantageous nanostructures.^41^, ^42^, ^43^ Hierarchical nanostructures have been confirmed as an effective and feasible approach to improve the performances of electrode materials.^44^, ^45^, ^46^ Hierarchical nanostructures usually include primary nanosized building blocks and second microarchitecture, which elicit synergistic effects on the improvement of the performances of electrode materials.^47^, ^48^ As promising electrode materials, hierarchically nanostructured TMOs possess numerous intrinsic outstanding features: (1) hierarchical nanostructures possess a high specific surface area, which can ensure a sufficient contact area between active electrode materials and electrolytes and provide more active sites for redox reactions;^49^, ^50^, ^51^ (2) the unique structure can shorten the diffusion pathway of Li ions and markedly improve the kinetic performance;^52^, ^53^, ^54^ (3) hierarchical architecture can serve as a buffer to accommodate severe changes in volume during lithiation/delithiation processes.^55^, ^56^, ^57^, ^58^

In this review, research progress on hierarchically nanostructured TMOs for high‐performance LIBs is mainly introduced. The structure, synthesis, and electrochemical performance of hierarchically nanostructured TMOs are systematically reviewed. Hierarchically nanostructured composites based on TMOs with carbon materials or conductive polymers are also summarized. Some personal perspectives are also presented to further develop LIBs based on hierarchically nanostructured TMOs.

It should be noted that the materials discussed in this review include only TMOs whose all metal elements belong to transition metal. Lithium TMOs, such as Li_4_Ti_5_O_12_,^59^, ^60^ LiCoO_2_,^61^, ^62^ and LiMn_2_O_4_,^63^, ^64^ which are of great value and importance to LIBs field, were not included in this review, as the lithium element does not belong to transition metal.

## 2 Hierarchical TMOs Based on Conversion Reaction

### 2.1 Iron Oxides

**Figure 3:**
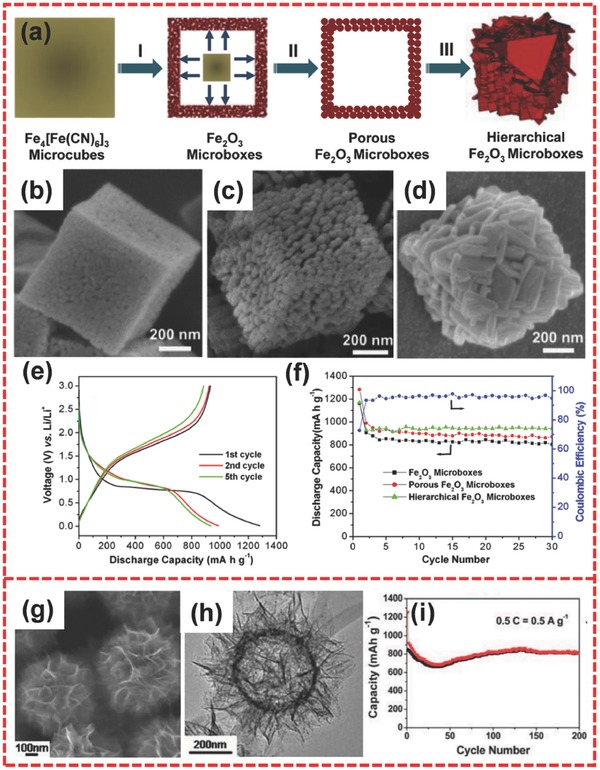
a) Schematic of the formation of hollow Fe_2_O_3_ microboxes and the evolution of the shell structure with the increasing calcination temperature. Field emission SEM (FESEM) images of hollow Fe_2_O_3_ microboxes obtained at b) 350 °C, c) 550 °C, d) 650 °C, respectively. e) Discharge/charge voltage profiles of porous Fe_2_O_3_ microboxes obtained at 550 °C. f) Cycling performance of Fe_2_O_3_ microboxes (350 °C), porous Fe_2_O_3_ microboxes (550 °C), and hierarchical Fe_2_O_3_ microboxes (650 °C) and Coulombic efficiency of porous Fe_2_O_3_ microboxes (550 °C) over the voltage range 0.01–3.0 V versus Li/Li^+^ at 200 mA g^−1^. g,h) FESEM image and transmission electron microscopy (TEM) image of hierarchical Fe_2_O_3_ hollow spheres composed of ultrathin nanosheets. i) Cycling performance of the hierarchical hollow spheres of Fe_2_O_3_ at 500 mA g^−1^. a–f) Reproduced with permission.^73^ Copyright 2012, American Chemical Society. g–i) Reproduced with permission.^76^ Copyright 2013, Royal Society of Chemistry.

Iron oxides, including Fe_2_O_3_ and Fe_3_O_4_, have been considered as promising candidates for the new generation of anode materials because of their abundant source, nontoxicity, high corrosion resistance, and low cost.^65^, ^66^, ^67^ Fe_2_O_3_ possesses a high theoretical specific capacity (1007 mA h g^−1^) on the basis of the conversion reaction mechanism: Fe_2_O_3_ + 6Li^+^ + 6e^−^ ↔ 2Fe + 3Li_2_O.^68^, ^69^ Hierarchically nanostructured Fe_2_O_3_ materials with various morphological characteristics have been investigated. For example, Cao et al. fabricated a hierarchical porous Fe_2_O_3_ nanosheets via an in situ approach.^70^ Lou and co‐workers reported the synthesis of hierarchical Fe_2_O_3_ hollow spheres by using a quasi‐emulsion‐templated method.^71^ Xu et al. also synthesized multishelled hierarchically hollow microspheres through sacrificial hard template method.^72^ All of these hierarchical materials exhibit good performances. In addition, Lou and co‐workers fabricated hierarchical Fe_2_O_3_ microboxes through a simple process of annealing prussian blue microcubes.^73^ This unique fabrication strategy provides a new route to produce uniform anisotropic hollow architectures compared with widely used solution‐based approaches.^74^, ^75^ As schematically depicted in **Figure** **3**a, the formation process of hierarchical Fe_2_O_3_ microbox involves three stages. Stage I occurred at 350 °C and assisted by outward gas flow, which can induce oxidative decomposition. As a result, Fe_2_O_3_ shell with a relative dense layer and a large inner cavity is formed. Fe_2_O_3_ microboxes were gradually converted into highly porous microboxes consisting of numerous Fe_2_O_3_ nanoparticles (NPs) because of crystal growth when temperature was increased to 550 °C (stage II). As temperature was further increased to 650 °C (stage III), porous Fe_2_O_3_ microboxes were transformed into a well‐defined hierarchical Fe_2_O_3_ microboxes composed of nanoplatelets. The corresponding scanning electron microscopy (SEM) images are shown in Figure 3b–d. In comparison to other reported Fe_2_O_3_‐based materials, hierarchical Fe_2_O_3_ microboxes delivered largely improved lithium storage properties. The porous Fe_2_O_3_ microbox sample (porous Fe_2_O_3_ obtained at 500 °C) exhibited a large reversible specific capacity of about 950 mA h g^−1^ (Figure 3e). Cycling performance was also evaluated, and all of the three samples exhibited good cycling stability. Compared with the Fe_2_O_3_ samples obtained at 350 and 500 °C, the hierarchical Fe_2_O_3_ microboxes obtained at 650 °C yielded an enhanced cycling performance over 30 cycles at 200 mA g^−1^ (Figure 3f).

Hierarchical hollow spheres have also been investigated as anode materials for LIBs. Zhu et al. fabricated hierarchical Fe_2_O_3_ hollow spheres through a template‐guided interfacial reaction method under hydrothermal conditions.^76^ These hollow spheres were constructed from the ultrathin nanosheets (Figure 3g,h). The nanosheets presented an average thickness of ≈3.5 nm and showed a favorable exposure of (110) facets. In the evaluation of the anode materials in LIBs, hierarchical hollow Fe_2_O_3_ spheres achieved a high reversible discharge capacity of 815 mA h g^−1^ after the 200th cycle and thus delivered excellent cycling stability during the charge/discharge process (Figure 3i).

**Figure 4:**
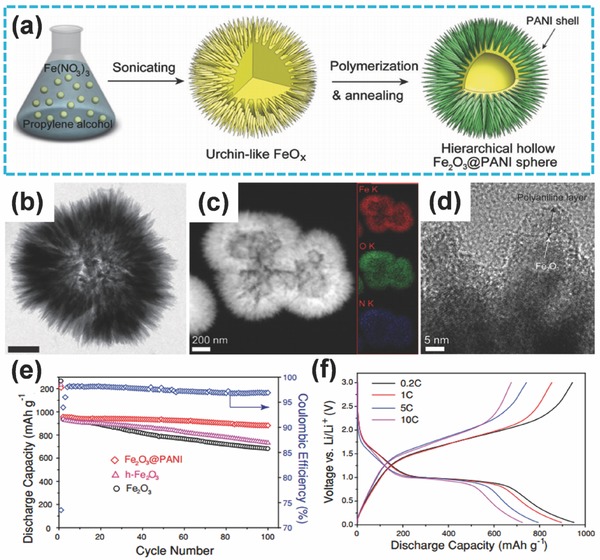
a) Schematic of the procedure to fabricate hierarchical Fe_2_O_3_@PANI through simultaneous interior and exterior constructions. b) TEM image of Fe_2_O_3_@PANI (Scale bar is 200 nm). c) High‐angle annular dark‐field scanning TEM (HAADF‐STEM) image of Fe_2_O_3_@PANI (inset images are EDS mapping of Fe, O, and N). d) HRTEM image of Fe_2_O_3_@PANI. e) Cyclic performance of Fe_2_O_3_, h‐Fe_2_O_3_, and Fe_2_O_3_@PANI electrodes at 0.1 C. f) Galvanostatic charge/discharge curves of Fe_2_O_3_@PANI electrode at different cycling rates. a–f) Reproduced with permission.^80^ Copyright 2013, Wiley‐VCH.

Considerable effort has been focused to further enhance the rate capability of LIBs. Coating of the conductive materials on TMOs is an effective strategy that solves the problem of the inherent poor electronic conductivity of iron oxides.^77^, ^78^, ^79^ Jeong et al. adopted this strategy to synthesize hierarchical hollow spheres of Fe_2_O_3_@polyaniline (PANI) via a template‐free sonochemical approach (**Figure** **4**a).^80^ Figure 4b,c demonstrates the urchin‐like hollow structure of the sample. As shown in Figure 4d, urchin‐like Fe_2_O_3_ was coated with the PANI shell with a thickness of around 5–10 nm. Thus, the conductivity of this architecture enhanced largely due to the improving of the electron transport in the coating polymer.^81^, ^82^ In addition, the hierarchical architecture could overcome the problem of large volume variations during the charge/discharge process. Thus, when evaluated as anode material for LIBs, the hierarchical hollow microsphere nanostructure of Fe_2_O_3_@PANI showed a better performance than hierarchical hollow Fe_2_O_3_ (h‐Fe_2_O_3_) and Fe_2_O_3_. Minimal capacity decay was observed during the cycling process, and a high capacity of 893 mA h g^−1^ could be maintained even after 100 cycles (Figure 4e). Meanwhile, as shown in Figure 4f, an excellent rate capability could be achieved.

**Figure 5:**
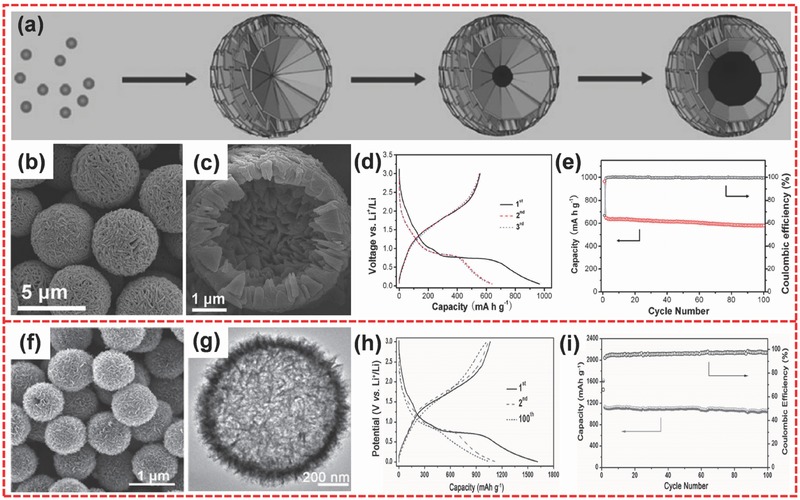
a) Schematic of the formation process of the hierarchical Fe_3_O_4_ hollow microspheres. b,c) FESEM images of hierarchical Fe_3_O_4_ hollow microspheres under different magnifications. d) Discharge/charge curves of Fe_3_O_4_ hollow microspheres at 200 mA g^−1^. e) Cycling performance of hierarchical Fe_3_O_4_ hollow microspheres at 200 mA g^−1^. f,g) SEM and TEM images of the highly uniform hierarchical Fe_3_O_4_ hollow spheres. h) Charge/discharge voltage profiles of hierarchical Fe_3_O_4_ hollow spheres for the first, second, and 100th cycles at 500 mA g^−1^. i) Cycling performance of hierarchical Fe_3_O_4_ hollow spheres at 500 mA g^−1^. a–e) Reproduced with permission.^92^ Copyright 2013, Wiley‐VCH. f–i) Reproduced with permission.^56^ Copyright 2015, Wiley‐VCH.

Fe_3_O_4_ has been widely used in LIBs as anode material. This material possesses a high theoretical capacity of 925 mA h g^−1^ according to the electrochemical conversion reaction: Fe_3_O_4_ + 8Li^+^ + 8e^−^ ↔ 3Fe + 4Li_2_O.^83^ In recent years, hierarchical hollow Fe_3_O_4_ has attracted considerable attention because of its excellent properties.^84^ Hierarchical hollow architecture can not only shorten the diffusion path of Li ions but also alleviate the stress caused by the volume change during the cycling process.^85^, ^86^, ^87^, ^88^ In general, hierarchical hollow structure is mostly prepared through the template method.^89^, ^90^, ^91^ Nevertheless, this method usually suffers from the serious problem of partial collapse in hollow structures after template removing. Moreover, the process is costly and tedious.^89^, ^90^ For this reason, Lou and co‐workers developed a facile solvothermal method to prepare hierarchical hollow microspheres composed of nanoplates (**Figure** **5**a–c).^92^ The product was formed through the self‐assembly of initial small nanoparticles and a subsequent inside‐out Ostwald ripening process.^93^ The product delivered a high initial discharge capacity of 960 mA h g^−1^ (Figure 5d). The hierarchical Fe_3_O_4_ nanostructure exhibited good cycling performance and a high reversible capacity of 580 mA h g^−1^ after 100 cycles at 200 mA g^−1^ (Figure 5e). Moreover, Lou and co‐workers further prepared the highly uniform hierarchical Fe_3_O_4_ hollow spheres (Figure 5f,g) through the solvothermal approach and a subsequent calcination.^56^ The hollow microspheres precursor was formed via a special self‐template process, which involves the initial formation of robust spheres and a subsequent chemical transformation into hollow spheres. Then, these hierarchical Fe_3_O_4_ hollow microspheres were obtained after annealing. Figure 5g shows that these hierarchical hollow spheres are constructed with ultrathin 2D nanoflakes. As we know, 2D ultrathin nanomaterials have received considerable attention because of their favorable properties of large specific surface area and short diffusion path.^55^, ^94^ Thus, this structure can enhance the performance of LIBs considerably. When evaluated as anode materials for LIBs, a high specific capacity of 1046 mA h g^−1^ could be retained after 100 cycles at 500 mA g^−1^, a high capacity retention of 94% can be obtained compared with the second cycle (Figure 5h,i). Notably, the unique hierarchical nanostructure can deliver a discharge capacity of up to 457 mA h g^−1^ even at a high current density of 10 A g^−1^, indicating a notable rate capability.

**Figure 6:**
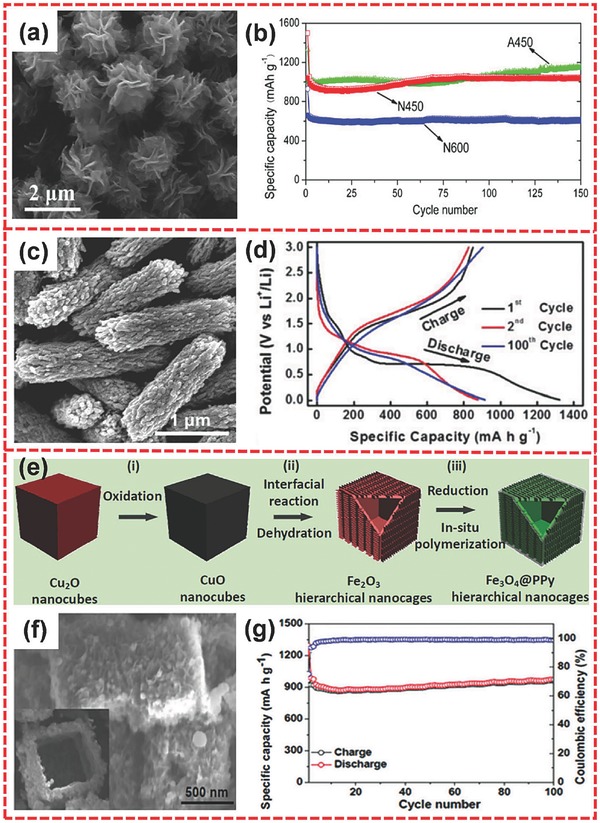
a) SEM image of hierarchical Fe_3_O_4_/C microflowers. b) Cycle performance of N450, N600, and A450 at 0.2 C. c) FESEM image of the sheaf‐like Fe_3_O_4_/C hierarchical microrods. d) Galvanostatic voltage profiles for the 1st, 2nd, and 100th cycles at 500 mA g^−1^. e) Schematic of the formation process for hierarchical Fe_3_O_4_@PPy nanocages. f) SEM image of Fe_3_O_4_@PPy nanocomposites. g) Cycling performance of hierarchical Fe_3_O_4_@PPy nanocages at 200 mA g^−1^. a,b) Reproduced with permission.^96^ Copyright 2011, Elsevier. c,d) Reproduced with permission.^98^ Copyright 2015, Royal Society of Chemistry. e–g) Reproduced with permission.^101^ Copyright 2016, Wiley‐VCH.

The construction of the nanocomposite of Fe_3_O_4_ with conductive materials is also studied to solve the deficiency of poor rate capability. Chen and co‐workers reported a nanostructure of 3D hierarchical carbon‐encapsulated Fe_3_O_4_ spheres, which could attain a capacity of 910 mA h g^−1^ after 600 cycles at 1 A g^−1^.^53^ Lu and co‐workers developed an easy and environmentally friendly approach to fabricate a hierarchical nanostructure of carbon‐decorated iron oxide microcuboids.^95^ The electrode material was obtained through the annealing treatment for iron metal–organic frameworks (MOFs) precursors. This product demonstrated a high reversible capacity of 975 mA h g^−1^ after 50 cycles at 0.1 A g^−1^. Jin et al. synthesized the hierarchical flower‐like Fe_3_O_4_/C nanocomposite via a facile strategy (**Figure** **6**a).^96^ The iron alkoxide precursor was prepared via a solvothermal reaction and then the final product was obtained through the controlled thermal decomposition of the as‐prepared precursor. The hierarchical nanostructure was assembled from porous nanoflakes composed of Fe_3_O_4_ nanoparticles and amorphous carbon. The samples obtained at 450 and 600 °C under nitrogen and at 450 °C in air were denoted as N450, N600, and A450, respectively. When tested for LIBs, the three samples all exhibited good cycling performance. In particular, A450 and N450 attained high capacities of about 1000 mA h g^−1^ at 0.2 C and outstanding cycling performances (Figure 6b). The excellent performance could be ascribed to the distinct merits of hierarchical nanocomposite architecture, which provided a robust structure and a high electronic conductivity. The carbon coating could not only increase conductivity but also play a role of physical barrier to hinder the aggregation of Fe_3_O_4_ nanoparticles during the cycling process.^97^ Lou and co‐workers also prepared a functional hierarchical nanomaterials of sheaf‐like Fe_3_O_4_/C nanocomposite consisting of porous nanowires (Figure 6c), which are obtained through a solvothermal process and a subsequent heat treatment.^98^ When tested as anode material for LIBs, the Fe_3_O_4_/C porous microrod sample exhibited excellent electrochemical lithium storage properties. Meanwhile, the product could achieve a high capacity of 1324 mA h g^−1^ at first cycle, and no fading occurred from the 2nd to 100th cycle, indicating good cycling stability (Figure 6d).

The strategies of forming the hierarchically hollow nanostructure and forming the nanocomposite of conductive materials and TMOs are two effective approaches to address the problems of low rate capability and poor cycling life.^99^, ^100^ Zhu and co‐workers combined the two advantages and prepared a hierarchical nanocage of Fe_3_O_4_@polypyrrole (PPy) (Figure 6f) through a Cu_2_O template‐assisted interfacial reaction together with a process of reduction and polymerization (Figure 6e).^101^ The electrochemical performances of the sample were significantly enhanced because of the synergistic effect of hierarchically hollow architecture and nanocomposite. The hierarchical nanocomposite could maintain a high specific capacity of about 950 mA h g^−1^ after 100 cycles at 200 mA g^−1^. Moreover, compared with the second cycle, minimal capacity fade occurred during the 100 cycles (Figure 6g).

### 2.2 Cobalt Oxides

**Figure 7:**
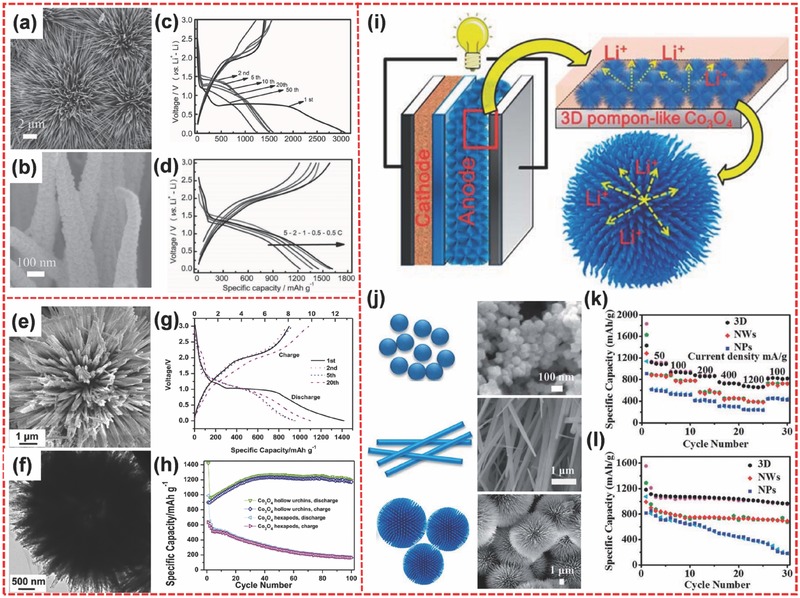
a) SEM image of hierarchical CoO nanowires viewed from the top. b) Magnified SEM image of several CoO nanowires. c) The charge/discharge curves of hierarchical CoO hierarchical nanowires at 1 C. d) The charge/discharge curves of hierarchical CoO hierarchical nanowires at various current densities. e,f) FESEM and TEM images for the hierarchical urchin‐like Co_3_O_4_ hollow spheres. g) Galvanostatic charge/discharge curves of hierarchical Co_3_O_4_ at 90 mA g^−1^. h) Cycling performances of hierarchical Co_3_O_4_ and Co_3_O_4_ hexapods at 0.1 C. i) Schematic of the lithium‐ion battery with hierarchical pompon‐like Co_3_O_4_ as anode material. j) SEM images of three Co_3_O_4_ materials with different morphologies: nanoparticles; nanowires; 3D pompon‐like porous spheres. k) Rate performance of three Co_3_O_4_ materials at different current densities. l) Cycling performance of three Co_3_O_4_ materials at 50 mA g^−1^. a–d) Reproduced with permission.^7^ Copyright 2015, Wiley‐VCH. e–h) Reproduced with permission.^117^ Copyright 2012, Elsevier. i–l) Reproduced with permission.^119^ Copyright 2014, Royal Society of Chemistry.

Cobalt oxides, including CoO and Co_3_O_4_, have drawn tremendous interest because of their excellent redox properties. CoO possesses a high theoretic specific capacity of 716 mA h g^−1^ based on the reaction: CoO + 2Li^+^ + 2e^−^ ↔ Co + Li_2_O.^102^, ^103^, ^104^ Jiao and co‐workers synthesized hierarchical CoO nanowire clusters (**Figure** **7**a) directly grown on the current collector through in situ synthesis.^7^ The nanowires were composed of ultrasmall nanoparticles (≈10 nm) (Figure 7b). In a typical electrode material, the integrity of the electrodes played a vital role in sustaining good cycle stability.^105^, ^106^ In general, for the fabrication of traditional electrodes, polymeric binders are utilized to ensure that active materials bonded on the current collector stably. However, this method often suffers from blocking of the pathways for lithium ion diffusion and decreasing electronic conductivity, resulting in poor capacity and rate capability. Binder‐free electrodes have been developed to solve these problems.^31^, ^107^ The electrode materials can stick to the current collector stability because of in situ tight adhesion. Meanwhile, among diverse in situ synthesis techniques, the hydrothermal method may be a facile, controllable, and feasible route.^108^, ^109^ When used as the LIB electrode, the binder‐free hierarchical CoO demonstrates excellent rate capability. The first discharge could achieve an ultrahigh capacity of 3087 mA h g^−1^ at 1 C rate, and subsequent cycle showed a reversible capacity of about 1580 mA h g^−1^ (Figure 7c). Even at a high rate of 5 C, a high capacity of 1330.5 mA h g^−1^ could be maintained (Figure 7d).

In comparison with CoO, Co_3_O_4_ has received more attention because Co_3_O_4_ possesses a markedly higher theoretical capacity of ≈890 mA h g^−1^.^110^, ^111^, ^112^, ^113^, ^114^ Tu and co‐workers reported a hierarchical porous Co_3_O_4_ nanostructure prepared using polystyrene sphere as the template by electrodeposition method. The sample could deliver 80% of the theoretical capacity at 1 C after 50 cycles.^115^ Li et al. prepared hierarchical Co_3_O_4_ micro/nanostructures with a star‐like morphology via a self‐assembly process. The capacity could remain at 995 mA h g^−1^ after 100 cycles at 500 mA g^−1^.^116^ Rui et al. prepared the hierarchical urchin‐like hollow Co_3_O_4_ microspheres assembled by 1D nanowires composed of plenty of interconnected Co_3_O_4_ nanoparticles via a template‐free hydrothermal method (Figure 7e,f).^117^ This unique hierarchical urchin‐like hollow material exhibited a high specific capacity and an excellent cycling performance (Figure 7g,h). Apart from the template‐free approach, the template‐assisted synthesis strategy was utilized by Chen et al. to synthesize urchin‐like hierarchical Co_3_O_4_ hollow spheres.^118^ In their work, hexadecyl trimethyl ammonium bromide was used as the template to prepare urchin‐like cobalt carbonate hydroxide hydrate hollow‐sphere precursor. The product was obtained after the thermal decomposition of the precursor. This hierarchical urchin‐like sample could deliver a superior lithium storage performance of 1342.2 mA h g^−1^ at 0.1 C rate.

In addition, a pompon‐like nanostructure of 3D hierarchical porous Co_3_O_4_ microspheres was prepared by Hao et al. through a hydrothermal approach.^119^ As shown in Figure 7j, three different Co_3_O_4_ samples of nanoparticles, nanowires, and 3D hierarchical pompon‐like spheres were prepared by controlling the hydrothermal reaction conditions. Evidently, 3D hierarchical pompon‐like Co_3_O_4_ spheres exhibited more favorable properties compared with the other structures. Figure 7i illustrates the possible Li ion transfer mode within the hierarchical pompon‐like Co_3_O_4_ material. The unique hierarchical architecture offered plenty of active sites to increase the contact area between electrolyte and electrodes and shortened the Li ion diffusion distance. Furthermore, the porous structure of the 3D frameworks could buffer the volume variation strain during the charge/discharge processes.^120^ Figure 7k,l shows the rate performance and cycling performance of three samples, respectively. The test results indicate that the 3D hierarchical pompon‐like Co_3_O_4_ architecture exhibits higher specific capacity and better cycle performance than Co_3_O_4_ nanoparticles and nanowires.

### 2.3 Nickel Oxides

**Figure 8:**
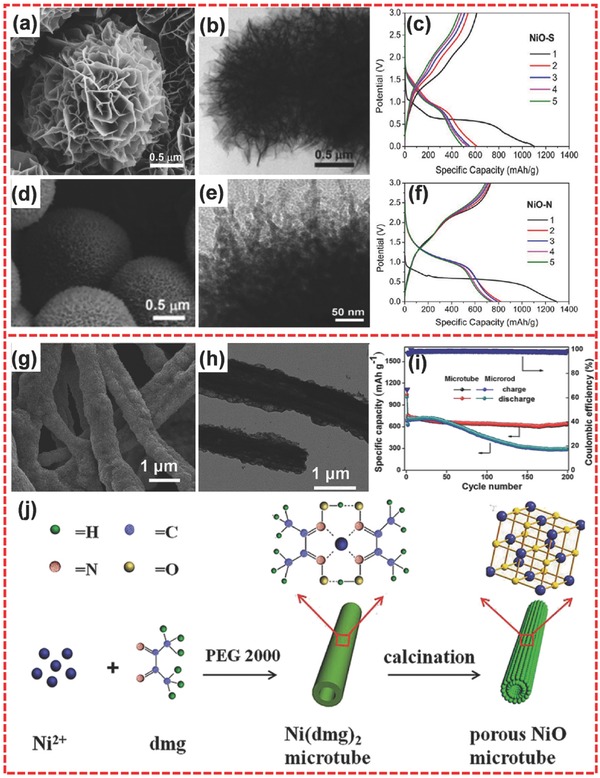
a,b) SEM and TEM images of flower‐like hierarchical NiO‐S microspheres consisting of interconnected nanoplates. c) Galvanostatic charge/discharge curves of NiO‐S at 50 mA g^−1^. d,e) SEM and TEM images of urchin‐like hierarchical NiO‐N microspheres consisting of radial nanowires. f) Galvanostatic charge/discharge curves of NiO‐N at 50 mA g^−1^. g,h) SEM and TEM images of hierarchical porous NiO microtubes. i) Cycling performances and coulombic efficiencies of porous NiO microtubes and NiO microrods at 1000 mA g^−1^. j) Schematic illustration of the formation of porous NiO microtubes. a–f) Reproduced with permission.^125^ Copyright 2013, American Chemical Society. g–j) Reproduced with permission.^128^ Copyright 2014, Royal Society of Chemistry.

NiO has been extensively utilized as a favorable anode material because of its high theoretical capacity of 718 mA h g^−1^ and high volumetric energy density.^121^, ^122^, ^123^ Chen et al. synthesized hierarchical porous NiO nanosheet arrays by a hydrothermal method.^124^ The hierarchical structure was highly porous and was composed of cross‐linked small‐sized nanoparticles. The sample delivers a specific capacity of 511 mA h g^−1^ at 3 A g^−1^, which is higher than that of the normal NiO nanosheet arrays (374 mA h g^−1^ at 3 A g^−1^). Wang and co‐workers prepared hierarchical NiO samples with two different morphologies: flower‐like hierarchical NiO‐S microspheres (**Figure** **8**a) and urchin‐like hierarchical NiO‐N microspheres (Figure 8d) through a solvothermal approach.^125^ Different morphologies were ascribed to different nickel sources. NiO‐S and NiO‐N came from NiSO_4_ and Ni(NO_3_)_2_, respectively. The hierarchical structure was obtained from self‐assembly, which had been a favorable strategy in the rational design of superior 3D hierarchical nanostructures.^126^, ^127^ Moreover, hierarchical NiO‐S and NiO‐N architectures were constructed from nanosheets (Figure 8b) and nanowires (Figure 8e), respectively. As anodes for LIBs, NiO‐S and NiO‐N possessed initial capacities of 1104 (Figure 8c) and 1295 mA h g^−1^ (Figure 8f) at 50 mA g^−1^, respectively. Meanwhile, little fading occurred for the two samples after the second cycle, thus, demonstrating their good cycling performance.

The hierarchical microtube structure has also been prepared for the electrode materials of LIBs. Yang and co‐workers successfully synthesized hierarchically porous NiO microtubes by simple precipitation method (Figure 8g,h).^128^ With the assistance of PEG 2000, Ni(dmg)_2_ (dmg = dimethyl‐glyoxime) gradually formed microtubes. Hierarchical NiO was obtained after calcination of Ni(dmg)_2_ microtubes (Figure 8j). When applied in LIBs, the hierarchically porous NiO microtubes achieved a high initial discharge capacity of 1180 mA h g^−1^ at 200 mA g^−1^. Meanwhile, the product could deliver a reversible capacity of 640 mA h g^−1^ after 200 cycles at 1000 mA g^−1^ and little capacity loss during 100 cycles (Figure 8i).

### 2.4 Manganese Oxides

Manganese oxides, including MnO, Mn_3_O_4_, Mn_2_O_3_, and MnO_2_, are promising alternative materials for LIBs because of their high theoretical capacity (756, 937, 1018, and 1230 mA h g^−1^ for above four manganese oxides, respectively) and thermal stability.^129^, ^130^, ^131^, ^132^, ^133^, ^134^ Manganese oxides also possess lower operating potentials compared with Fe‐, Co‐, and Ni‐based TMOs.^135^, ^136^ In previous reports, Mn_2_O_3_ has received more attention than other manganese oxides.^137^, ^138^ Mn_2_O_3_ nanostructured materials with different morphologies, such as nanorods,^139^ nanospheres,^140^ microporous particles,^141^ and hollow microspheres,^138^ have been reported. Recently, Su and co‐workers reported 3D bicontinuous hierarchically porous Mn_2_O_3_ single crystals (BHP‐Mn_2_O_3_‐SCs) utilized in LIBs.^142^ When cycled at current densities of 50 and 1000 mA g^−1^, the reversible capacities of the product were about 910 and 600 mA h g^−1^, respectively. Yang and co‐workers synthesized hierarchical Mn_2_O_3_ microspheres through a rapid microwave method, achieving a specific capacity of 525 mA h g^−1^ after 500 cycles at 1000 mA g^−1^.^143^ Sun and co‐workers prepared hierarchical hollow Mn_2_O_3_ microspheres,^137^ which delivered a reversible capacity of 580 mA h g^−1^ after 140 cycles at 500 mA g^−1^. Furthermore, Chen and co‐workers designed a multistep strategy in the synthesis of hierarchical porous double‐layered Mn_2_O_3_ hollow microspheres containing porous inner and outer shells that consisted of plenty of tiny nanoparticles.^138^ For this special architecture, numerous active sites were obtained as a result of sufficient electrode/electrolyte contact area. Thus, hierarchical porous Mn_2_O_3_ material exhibited good performance even at a high current density. It delivered a reversible discharge capacity of 471 mA h g^−1^ after 100 cycles at a relatively high current density of 3200 mA g^−1^.

**Figure 9:**
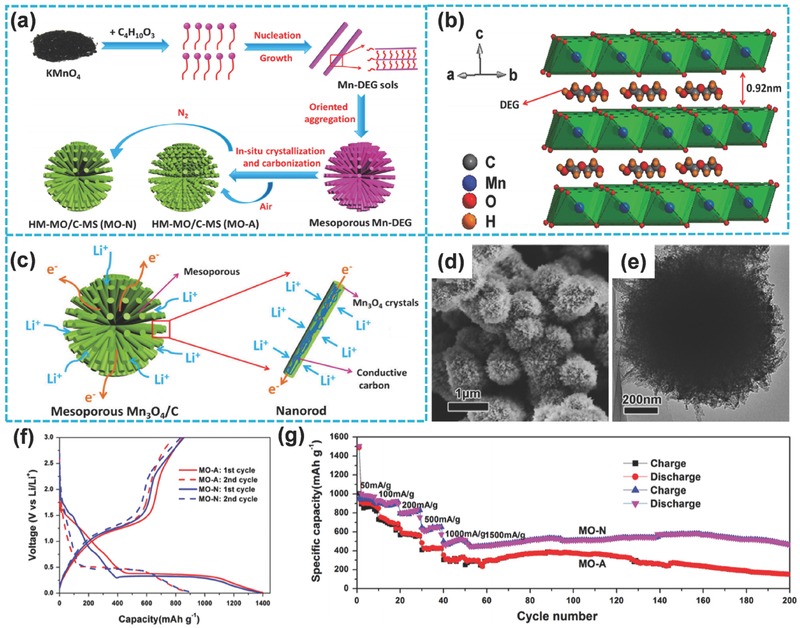
a) Schematic illustration of HM‐MO/C‐MS synthesis. b) The crystal structure of Mn‐DEG. c) Schematic illustration of lithium insertion mechanism in HM‐MO/C‐MS. d,e) SEM and TEM images of the MO‐N sample. f) First and second charge/discharge profiles at 100 mA g^−1^ under a voltage range of 0.01–3 V. g) Charge/discharge capacities at various current densities and cycling performance at 1500 mA g^−1^. a–g) Reproduced with permission.^146^ Copyright 2015, Elsevier.

Aside from Mn_2_O_3_, Mn_3_O_4_ also has good electrochemical properties among manganese oxides.^144^, ^145^, ^146^, ^147^ Nevertheless, pure Mn_3_O_4_ material suffers from poor electrical conductivity and inferior cycle stability and demonstrates low lithiation activity.^148^ Recently, Su and co‐workers prepared 3D hierarchical urchin‐like Mn_3_O_4_/C microspheres through solution‐phase reaction and subsequent carbonization (**Figure** **9**a–e),^146^ addressing the above issues to a great extent.^149^, ^150^ This special 3D hierarchical nanostructure could provide numerous accessible electroactive sites, shorten the transport distance for Li ions, and suppress volume changes during the reaction.^151^ At the same time, the conductivity of electrode materials is obviously improved from the conductive carbon in Mn_3_O_4_/C composites.^152^ Thus, this unique hierarchical architecture exhibited excellent electrochemical performances. With different heat‐treatment atmospheres, two types of hierarchical mesoporous Mn_3_O_4_/C microspheres were obtained. The products were denoted as MO‐A and MO‐N from the heat‐treatment at air and nitrogen atmosphere, respectively. As shown in Figure 9f, both samples exhibited a specific capacity of about 900 mA h g^−1^ at 0.1 A g^−1^ at the second cycle. In addition, the MO‐N sample demonstrated excellent rate capability and displayed specific capacities of 980, 900, 810, 650, and 510 mA h g^−1^ when cycling at different current densities of 50, 100, 200, 500, and 1000 mA g^−1^, respectively (Figure 9g). MO‐N sample also showed 93.7% capacity retention after 200 cycles at 1500 mA g^−1^ (Figure 9g).

### 2.5 Copper Oxides

As a promising TMO, CuO has also attracted numerous attention due to its high theoretical capacity (674 mA h g^−1^) and low toxicity.^153^, ^154^, ^155^ Hu et al. reported a hierarchical CuO octahedral structure by treating copper MOF template.^156^ The hierarchical CuO inherited the morphology of the Cu‐MOF precursor and was composed of many ultrathin nanosheets. When used as the anode material of LIBs, the sample exhibited a high reversible capacity of 836 mA h g^−1^ in the second cycle at 0.1 A g^−1^ and superb high‐rate performance (about 470 mA h g^−1^ at 4 A g^−1^). Additionally, capacity retention of nearly 100% can be realized after 400 cycles at 2 and 5 A g^−1^.

### 2.6 Mixed TMOs

**Figure 10:**
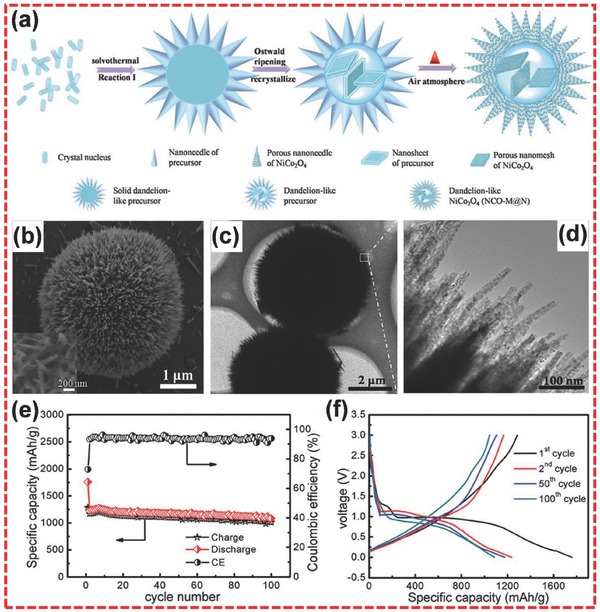
a) Schematic illustration of the formation of the dandelion‐like NCO‐M@N. b) SEM image of NCO‐M@N (the inset is used to describe the detail of the nanoneedles). c) The low‐magnification TEM image of NCO‐M@N. d) The magnified TEM image of the external nanoneedles (the selected area of the white box in (c). e) The charge/discharge capacities and the corresponding coulombic efficiency of the NCO‐M@N electrode at 200 mA g^−1^. f) Galvanostatic charge/discharge curves in the voltage range of 0.01–3.0 V at 200 mA g^−1^. a–f) Reproduced with permission.^160^ Copyright 2015, Royal Society of Chemistry.

Mixed TMOs, such as NiCo_2_O_4_ 157, 158 and MnCo_2_O_4_,^159^ have also been investigated as anode materials in LIBs. Compared to single TMOs, mixed TMOs usually display better electrochemical performance owing to the synergic effects of multiple metal species.^28^ Moreover, mixed TMOs usually exhibit better electrical conductivity.^157^, ^160^ For instance, NiCo_2_O_4_ shows higher electrical conductivity than nickel oxide and cobalt oxide.^161^ Guo et al. reported an effective method of simultaneously coordinating etching and precipitation reactions to prepare hierarchically hollow crossed NiCo_2_O_4_ nanocubes.^162^ When applied in LIBs, the product delivered a high reversible capacity of 1160 mA h g^−1^ at 200 mA g^−1^. Meanwhile, a high capacity retention of 91.1% could be obtained even after 200 cycles. Liu et al. fabricated hierarchical dandelion‐like NiCo_2_O_4_ microspheres@nanomeshes (NCO‐M@N) through a solvothermal method.^160^ As shown in **Figure** **10**a, the synthesis process undergone several stages to form the final dandelion‐like NiCo_2_O_4_. SEM and TEM images (Figure 10b,c) show that the sample has the dandelion‐like structure with numerous nanoneedles radially grown on the surface. Moreover, Figure 10d shows that nanoneedles consist of considerable small nanoparticles and a large portion of mesoporous structure, indicating that the product was a hierarchical structure. When used in LIBs, the sample showed a high reversible specific capacity of 1235.4 mA h g^−1^ at 200 mA g^−1^ (Figure 10e), a good cycling performance (capacity retention of 88% after 100 cycles) (Figure 10e), and a remarkable rate performance (785 mA h g^−1^ at 2 A g^−1^). It is worth noting that this material exhibited a small voltage hysteresis between charge curve and discharge curve (Figure 10f).

A comparison of typical hierarchically nanostructured TMOs based on conversion reaction is given in **Table** **1**.

**Table 1:** Electrochemical performances of various hierarchically nanostructured TMOs based on conversion reaction

Materials	Volume expansion percentage^163^	Feature	Electrochemical performancea)						Ref.
			Current density [mA g^−1^]	Capacity (initial cycle/second cycle) [mA h g^−1^]	Cycle number	Capacity retention [mA h g^−1^]	Initial coulombic efficiency	Average charge potential/average discharge potentialb) [v]	
Fe_2_O_3_	≈93.5%	Fe_2_O_3_ microboxes	200	1180/940	30	945	≈72%	≈1.68/≈0.875	73
		Hollow Fe_2_O_3_ spheres	500	1255/920	200	815	≈67%	≈1.8/≈0.875	76
		Hollow spheres of Fe_2_O_3_@ PANI	0.1 C	1208/≈950	100	893	73.5%	≈1.75/≈0.95	80
Fe_3_O_4_	≈80.4%	Hollow microspheres of Fe_3_O_4_	200	960/≈650	100	580	≈68%	≈1.75/≈0.79	92
		Highly uniform Fe_3_O_4_ hollow sphere	500	1614/1063	100	1046	66%	≈1.75/≈0.75	56
		Flower‐like Fe_3_O_4_/C nanocomposite	0.2 C	≈1500/≈1000	80	≈1000	74%	≈1.7/≈0.95	96
		Sheaf‐like Fe_3_O_4_/C nanocomposite	500	1324/≈920	200	837	64.1%	≈1.7/≈0.875	98
		Fe_3_O_4_@PPy nanocage	200	≈1300/≈1000	100	950	≈75%	≈1.65/≈0.625	101
CoO	≈84.8%	CoO nanowire clusters	1 C	3087/1580	50	1250	≈60%	≈2/≈1.08	7
Co_3_O_4_	≈102.2%	Urchin‐like Co_3_O_4_ hollow microspheres	0.1 C	1420/1100	100	≈1190	≈63%	≈2/≈1	117
		Pompon‐like porous Co_3_O_4_ microspheres	50	1552/≈1200	30	≈1000	≈77%	–	119
NiO	≈93.5%	Urchin‐like NiO microspheres	50	1295/≈800	5	≈720	≈55%	≈2.1/≈1	125
		Porous NiO microtubes	1000	≈1050/≈750	200	≈640	67.3%	≈2/≈1.08	128
Mn_2_O_3_	≈89.1%	BHP‐Mn_2_O_3_‐SCs	100	1473/≈950	50	845	64.3%	≈1.37/≈0.49	142
Mn_3_O_4_	≈68.1%	Urchin‐like Mn_3_O_4_/C microspheres	100	≈1400/≈900	50	915	66.5%	≈1.35/≈0.49	146
CuO	≈73.9%	CuO octahedral structure	100	1334.7/≈880	100	≈1100	≈63%	≈2.25/≈0.9	156
NiCo_2_O_4_	–	Hollow crossed NiCo_2_O_4_ nanocubes	100	1360/≈1150	200	1056	85.3%	≈1.65/≈0.75	162
	–	Dandelion‐like NiCo_2_O_4_	200	1760/≈1250	100	1086	73%	≈0.9/≈0.85	160

^a)^ It should be noted that, in addition to the performance shown in the table, the voltage efficiency and energy efficiency are also very important for the full cells assembled by these anode materials.^15^ These two performances need to be focused on as well in future research work

^b)^ The average charge/discharge potential is defined as the potential where half of the charge/discharge capacity has been reached.^15^

## 3 Hierarchical TMOs Based on Intercalation/Deintercalation Reaction

### 3.1 Titanium Dioxide

TiO_2_ has received numerous attention as an anode in LIBs because of its environmental friendliness, low cost, superior chemical stability, and improved safety.^164^, ^165^, ^166^, ^167^, ^168^ In comparison with the conversion reaction, the reaction mechanism of TiO_2_ is based on intercalation/deintercalation: TiO_2_ + *x*Li^+^ + *x*e^−^ ↔ Li*_x_*TiO_2_.^169^, ^170^, ^171^ In this reaction, TiO_2_ can host Li^+^ to generate Li*_x_*TiO_2_ instead of reducing to elemental titanium. Consequently, the theoretical specific capacity is naturally lower than those of TMOs based on the conversion reaction. However, and for exactly that reason, TiO_2_ holds the advantage of structural stability. Moreover, TiO_2_ possesses a merit of safety because of its relatively high lithium insertion/extraction operation voltage of 1.5–1.8 V, which may avoid the formation of solid electrolyte interface layers during electrochemical cycling.^172^, ^173^, ^174^ In general, TiO_2_, which can be used as an electrode of LIBs, mainly includes anatase TiO_2_, rutile TiO_2_, and TiO_2_‐B (bronze).^35^, ^170^

#### 3.1.1 Anatase TiO_2_

**Figure 11:**
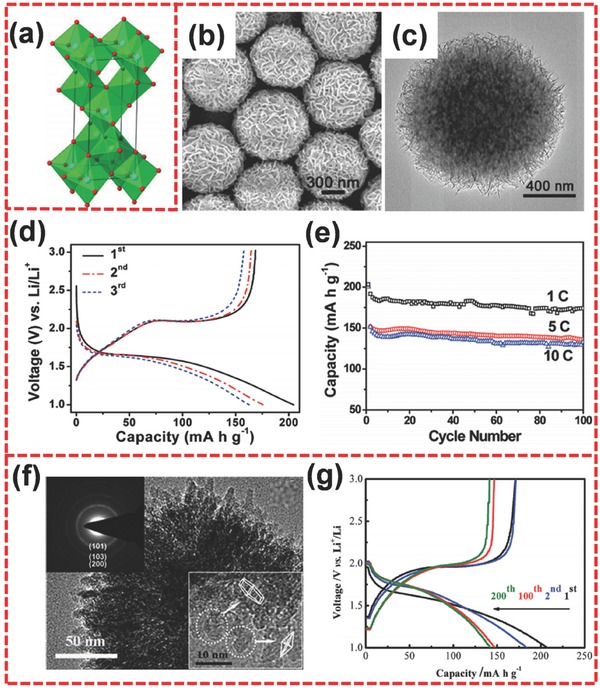
a) Crystallographic representation of anatase TiO_2_. b,c) SEM and TEM images of hierarchical anatase TiO_2_ spheres. d) Charge/discharge curves at 5 C (850 mA g^−1^) for the initial three cycles. e) Cycling performance at different rates. f) TEM image of anatase TiO_2_ microsphere. g) Charge/discharge profiles for different cycles at 10 C. a) Reproduced with permission.^35^ Copyright 2013, American Chemical Society. b–e) Reproduced with permission.^169^ Copyright 2010, American Chemical Society. f,g) Reproduced with permission.^185^ Copyright 2014, Royal Society of Chemistry.

Anatase TiO_2_ is generally the most electroactive host of Li insertion and has been the most widely investigated among the three kinds of TiO_2_ (**Figure** **11**a).^35^, ^175^, ^176^ Anatase TiO_2_ could accommodate 0.5 Li (Li_0.5_TiO_2_) and has a theoretical specific capacity of 167.5 mA h g^−1^.^169^, ^177^ Lou and co‐workers designed 3D hierarchical TiO_2_ nanocrystal microspheres composed of nanosheets (Figure 11b,c) through a facile approach,^169^ where the hierarchical nanostructure could exhibit outstanding performance because of the naked high‐energy facets. In previous reports, Yang et al. first synthesized anatase TiO_2_ microcrystals with ≈47% exposed (001) facets.^168^ Later, many researchers paid huge attention to study these microcrystals, resulting in exposed (001) facets that are as high as 89%.^178^, ^179^, ^180^, ^181^, ^182^ Lou and co‐workers also changed the strategy by reducing the thickness in the (001) direction and increasing the 2D lateral size of the (001) planes, thus, achieving nearly 100% exposed (001) facets and an increase in the specific surface area. These exposed facets do not only exhibit high reactivity in accelerating chemical reactions, but also shorten the transport path in the (001) direction, ensuring the excellent electrochemical performance for high‐power LIBs.^183^, ^184^ Furthermore, the nanosheets arranged into 3D hierarchical microspheres through self‐assembly. Figure 11d presents the charge/discharge curves of hierarchical TiO_2_ sphere electrode for the first few cycles at 5 C. A reversible capacity of 174 mA h g^−1^ remained after 100 charge/discharge cycles at 1 C (Figure 11e). Wei and co‐workers prepared hierarchically porous anatase TiO_2_ microspheres under a one‐step synthetic approach without using surfactants.^185^ This method could avoid the alterations of surface chemistry during removal of surfactants from the final product. These hierarchically porous anatase TiO_2_ microspheres consisted of ultrafine nanorods in the radial direction. The ultrafine nanorods contained tiny‐sized octahedral crystals (Figure 11f). The sample displayed a capacity of 142.3 mA h g^−1^ even after 200 cycles at 10 C (Figure 11g), indicating its excellent rate performance and good cycling stability. In addition, the strategy of MOFs template was also used to fabricate anatase TiO_2_ microspheres with hierarchical porous structure.^174^ The synthesis process includes the hydrolysis of titanium MOFs precursor and the subsequent calcination in air. Because MOFs serve as the template, the product contains considerable mesopores and macropores, which can increase the specific surface area of the material and can shorten the diffusion path of Li^+^ ions.^186^, ^187^ As a result, an excellent rate capability and outstanding cycling performance could occur when anatase TiO_2_ functioned as the anode for LIBs. The specific discharge capacity was about 155 mA h g^−1^ at 5 C. Meanwhile, capacity loss was about 6.5% after 200 cycles at 5 C rate in comparison with the second cycle.

#### 3.1.2 TiO_2_(B)

**Figure 12:**
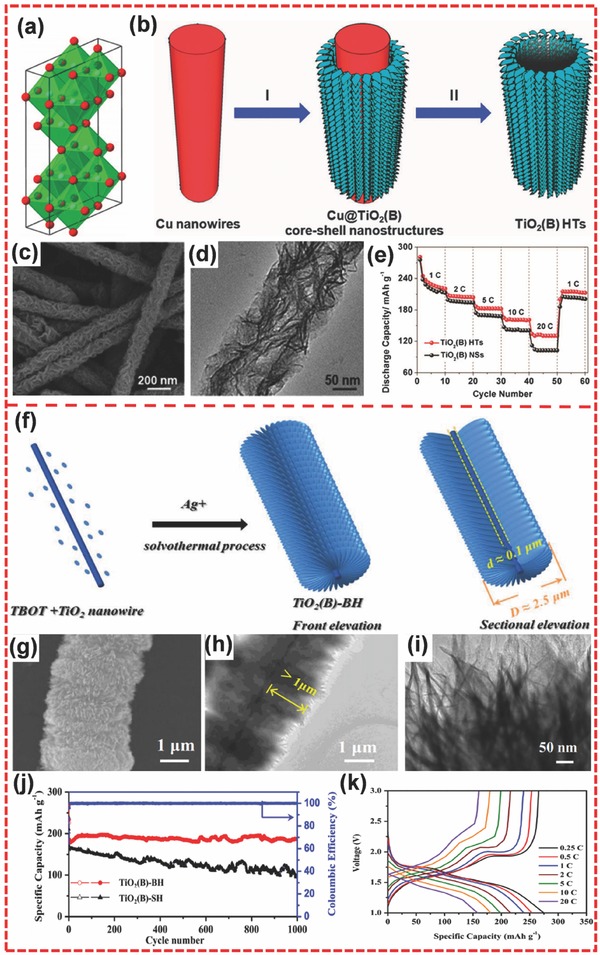
a) Crystallographic representation of TiO_2_(B). b) Schematic illustration of template‐assisted formation of TiO_2_(B) hierarchical tubes (TiO_2_(B) HTs). c,d) SEM and TEM images of TiO_2_(B) HTs. e) Cycling performance of TiO_2_(B) HTs and TiO_2_(B) NSs at various current rates. f) Illustration of TiO_2_(B)‐BH synthetic process. g) SEM image of TiO_2_(B)‐BH. h,i) TEM images of TiO_2_(B)‐BH at different magnifications. j) Comparative cycle performance of TiO_2_(B)‐BH and TiO_2_(B)‐SH at C/4 for the first, C/2 for the second, and 5 C for the rest of the cycles. k) The charge/discharge curves of TiO_2_(B)‐BH at various current densities. a) Reproduced with permission.^35^ Copyright 2013, American Chemical Society. b–e) Reproduced with permission.^197^ Copyright 2015, Royal Society of Chemistry. f–k) Reproduced with permission.^188^ Copyright 2016, Elsevier.

TiO_2_(B) is composed of corrugated sheets of edge‐ and corner‐shared TiO_6_ octahedra, which form an open framework structure.^35^ It has a monoclinic structure and possesses a lower density (3.7 g cm^−3^) than anatase (3.89 g cm^−3^) and rutile (4.25 g cm^−3^).^35^ Among various polymorphs of TiO_2_, TiO_2_(B) is a superior host for Li intercalation (**Figure** **12**a) because it possesses more open channels in the lattice than anatase and rutile.^188^, ^189^ Therefore, TiO_2_(B) has a higher theoretical capacity (335 mA h g^−1^) than other TiO_2_ polymorphs.^190^, ^191^ TiO_2_(B) is usually obtained through ion exchange from layered titanate or hydrothermal treatment of amorphous TiO_2_ in alkali and subsequent dehydration.^192^, ^193^, ^194^ Recently, Liu et al. reported a one‐step preparation for hierarchical porous TiO_2_(B) constructed by nanosheets with 5–10 nm thickness,^195^ and the sample could deliver a specific capacity of 216 mA h g^−1^ at 10 C. Su and co‐workers also synthesized hierarchical porous TiO_2_(B) spheres,^196^ achieving a specific capacity of 221 mA h g^−1^ at 10 C. Lou and co‐workers developed a solvothermal strategy assisted by a Cu nanowire template for the synthesis of TiO_2_(B) with hierarchical hollow tubular structure (TiO_2_(B) HTs) (Figure 12b).^197^ As shown in Figure 12c,d, these hierarchical tubular architectures consisted of TiO_2_(B) nanosheets. During preparation, TiO_2_(B) nanosheets were directly grown on the Cu nanowire template, and the template was then removed through the following solvothermal reaction. The resulting hierarchical TiO_2_(B) tubes showed high specific surface area and good shell permeability, delivering capacities of 216, 202, 182, 160, and 130 mA h g^−1^ when cycling at 1, 2, 5, 10, and 20 C, respectively (Figure 12e). Moreover, decreasing the current rate back to 1 C could retain a capacity of 210 mA h g^−1^. Liu et al. reported the synthesis of mesoporous TiO_2_(B) microspheres from combining template‐assisted ultrasonic spray pyrolysis with reflux, ion‐exchange, and heat treatment.^190^ The sample exhibited better performance than TiO_2_ (anatase) nanopowder when evaluated under the same conditions. At the relatively low rate of 0.1 C, the hierarchical material attained a discharge capacity of 311 mA h g^−1^. Meanwhile, the sample also displayed excellent rate performance, achieving specific capacities of 165, 130, and 115 mA h g^−1^ at 10, 30, and 60 C, respectively. Recently, Li et al. used tetrabutyl titanate, 1D TiO_2_ nanowires, and Ag^+^ to fabricate a TiO_2_(B) bunchy hierarchical structure (TiO_2_(B)‐BH) through solvothermal method (Figure 12f–i).^188^ In the absence of TiO_2_ nanowires, the TiO_2_(B) nanosheets aggregated into microspheres to form a TiO_2_(B) microsphere hierarchical structure (TiO_2_(B)‐SH). Because of the 1D TiO_2_ nanowire core, the bunchy hierarchical structure can maintain well in a long‐term cycling process. When evaluated as an anode for LIBs, TiO_2_(B)‐BH electrode exhibited better cycling performance than TiO_2_(B)‐SH, where the former could maintain a specific capacity of 186 mA h g^−1^ during 1000 cycles at 5 C, resulting in a capacity retention that is as high as about 100% (Figure 12j). TiO_2_(B)‐BH also exhibited a good rate performance, achieving a specific capacity of about 160 mA h g^−1^ at a rate of 20 C (Figure 12k).

#### 3.1.3 Rutile TiO_2_

Because of poor Li intercalation, the electrochemical performance of rutile TiO_2_ tends to be inferior compared with anatase TiO_2_ and TiO_2_(B). According to previous reports, bulk rutile TiO_2_ can only host <0.1 Li for every TiO_2_ unit at room temperature.^198^, ^199^ To solve this problem, rationally designed materials with hierarchical nanostructure have been prepared. Maier and co‐workers reported that nanosized rutile TiO_2_ could reversibly insert 0.5 Li to form Li_0.5_TiO_2_, which is comparable with anatase TiO_2_.^200^ Furthermore, Zhang and co‐workers synthesized novel hierarchical porous rutile TiO_2_ microspheres composed of nanorods and enhanced the intercalated Li amount up to 0.73 (Li_0.73_TiO_2_).^198^ These hierarchical rutile TiO_2_ microspheres achieved a specific capacity of 246 mA h g^−1^ in first discharge process at 0.1 C. Moreover, a good cycling performance could be obtained by delivering a capacity of 192 mA h g^−1^ after 30 cycles at 0.1 C. In comparison with the capacity of the second cycle (225 mA h g^−1^), only a few capacity was lost.

#### 3.1.4 TiO_2_/Carbon Composites

**Figure 13:**
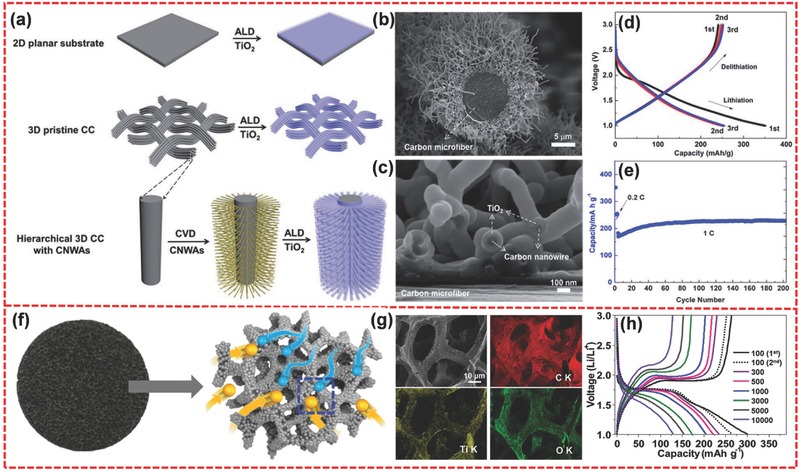
a) Schematic comparison and fabrication processes of TiO_2_ coating on a 2D planar substrate (first row), 3D pristine CC (second row), and hierarchical 3D CC with CNWAs (third row). b) Low magnification side view SEM image of the hierarchical TiO_2_@C core–shell structure. c) High magnification side view SEM image of the root of the TiO_2_@C core–shell structure. d) Initial three voltage profiles of the hierarchical TiO_2_@C core–shell electrode at 0.2 C. e) Capacity retention of the hierarchical TiO_2_@C core–shell electrode at 0.2 C for initial three cycles and then 1 C for subsequent 200 cycles. f) Digital photograph of the PG and a schematic structure of TiO_2_ NP@PG (light blue sphere: electron; yellow sphere: Li ion; light gray sphere: TiO_2_ NP; dark gray foam: graphene network foam). g) SEM image and EDS mapping images of TiO_2_ NP@PG. h) Voltage profiles of TiO_2_ NP@PG at various current densities. a–e) Reproduced with permission.^205^ Copyright 2015, Royal Society of Chemistry. f–h) Reproduced with permission.^206^ Copyright 2016, Wiley‐VCH.

To further enhance the electrochemical performance of TiO_2_, TiO_2_/carbon composites have also been studied.^201^, ^202^, ^203^, ^204^ Wang et al. reported the synthesis of 3D hierarchical TiO_2_@C core–shell structure as anode material for LIBs.^205^ As shown in **Figure** **13**a, carbon nanowire arrays (CNWAs) were first grown on carbon cloth (CC) through a thermal chemical vapor deposition method. Then TiO_2_ was coated on the carbon nanowires conformally utilizing the atomic layer deposition technique. SEM images showed that TiO_2_ was uniformly coated on the carbon nanowires (Figure 13b,c). The as‐prepared material could achieve excellent electrochemical performance. The initial discharge and charge capacities were as high as 351 and 241 mA h g^−1^ at 0.2 C, respectively (Figure 13d). The capacity could be stabilized at around 228 mA h g^−1^ after 200 cycles at 1 C (Figure 13e). Furthermore, long‐term cycling performance test indicated that the electrode achieved a remarkable cycling stability (only 15.2% capacity fading after 8000 cycles at 10 C).

Lee et al. fabricated a hierarchical structure of ultrafine TiO_2_ NP (about 6 nm) with macroporous graphene (PG) network foam (TiO_2_ NP@PG) (Figure 13f).^206^ Both macroporous open channels of PG and mesoporous structure of TiO_2_ nanocrystals could accelerate ionic diffusion during the reaction (Figure 13g). Meanwhile, this unique structure was also acting as the binder‐free electrode contacting with a current collector directly, leading to a rapid electronic transfer. When evaluated as anode material, this structure delivered an excellent electrochemical performance. It can achieve a large specific capacity of 262 mA h g^−1^ for the second cycle at 100 mA g^−1^ (Figure 13h). Even at an ultrahigh current rate of 10 A g^−1^, a specific capacity of 130 mA h g^−1^ could be obtained (Figure 13h), indicating a superior rate capability. Furthermore, this structure possessed unprecedented cycle stability, with 100% capacity retention over 10 000 cycles.

### 3.2 Niobium Oxides

**Figure 14:**
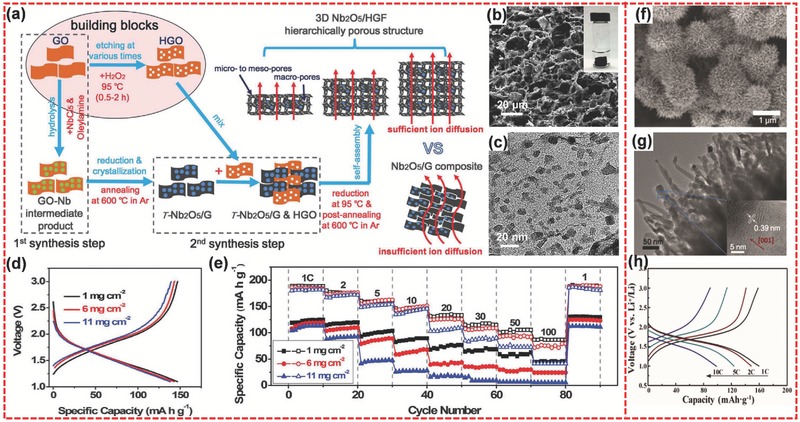
a) Illustration of the two‐step process flow to prepare 3D hierarchically porous composite architecture. b) Cross‐sectional SEM image of Nb_2_O_5_/HGF composite (inset: a free‐standing monolithic composite used to make the electrode). c) TEM image of graphene sheets with uniformly decorated Nb_2_O_5_ nanoparticles. d) Galvanostatic charge/discharge curves for the Nb_2_O_5_/HGF electrode at 10 C for the mass loadings of 1, 6, and 11 mg cm^−2^. e) Comparison of the rate performance between 1 and 100 C for Nb_2_O_5_/HGF (open) and Nb_2_O_5_/G (solid) electrodes under different mass loadings (1, 6, and 11 mg cm^−2^). f) FESEM image, and g) high‐magnification TEM image of urchin‐like Nb_2_O_5_ microspheres. The inset of (g) shows the HRTEM lattice image of a typical Nb_2_O_5_ nanorod. h) The initial discharge/charge curves of Nb_2_O_5_ microspheres at different rates from 1 to 10 C. a–e) Reproduced with permission.^209^ Copyright 2017, Science. f–h) Reproduced with permission.^210^ Copyright 2017, Royal Society of Chemistry.

Niobium oxides have emerged as prominent materials for LIBs.^207^, ^208^ Among various niobium oxides, Nb_2_O_5_ is the most thermodynamically stable species.^207^ Recently, Duan and co‐workers reported a 3D hierarchical nanostructure of Nb_2_O_5_/holey‐graphene framework (Nb_2_O_5_/HGF) for ultrahigh‐rate energy storage.^209^] **Figure** **14**a shows the detailed synthesis process of the Nb_2_O_5_/HGF sample and its structural characteristics. In this special structure, the interconnected graphene network accelerates the electron transport, and the hierarchical porous structure facilitates ion transport. SEM and TEM images (Figure 14b,c) show that the as‐prepared sample possesses the hierarchically porous structure and the graphene sheets are coated uniformly with the Nb_2_O_5_ nanoparticles. Notably, this material can realize high areal capacity and high‐rate capability at high mass loading. Figure 14d shows that only a relatively small voltage drop and capacity loss can be observed as increasing the mass loading of the Nb_2_O_5_/HGF electrode from 1 to 11 mg cm^−2^. Meanwhile, the Nb_2_O_5_/HGF electrode shows much less capacity loss for the high mass loading at ultrahigh current density compared with Nb_2_O_5_/GF electrode (Figure 14e). In addition, the capacity retention is 90% after 10 000 cycles at 10 C, and Coulombic efficiency is above 99.9%. This work represents an essential step for the practical application of electrochemical energy storage devices.

Liu et al. reported the synthesis of urchin‐like hierarchical Nb_2_O_5_ microspheres via a simple solvothermal approach and a subsequent thermal treatment.^210^ As shown in Figure 14f,g, Nb_2_O_5_ microspheres are composed of nanorods with an average diameter of 20 nm. When utilized as anode material for LIBs, the sample displayed the discharge capacity of 159.7, 148.5, 123.7, and 98.5 mA h g^−1^ at the rate of 1, 2, 5, and 10 C, respectively, indicating a good rate performance (Figure 14h). In addition, the capacity retention was 81% after 500 cycles at a high rate of 5 C.

### 3.3 Vanadium Pentoxide

**Figure 15:**
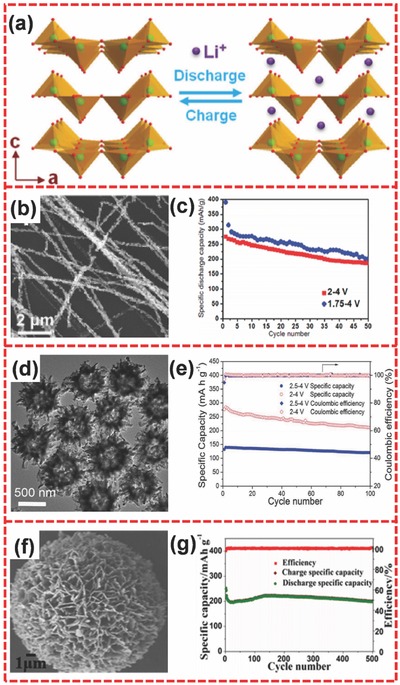
a) Schematic illustration of layered V_2_O_5_. b) SEM image of ultralong hierarchical V_2_O_5_ nanowires. c) Cycling performance of ultralong hierarchical V_2_O_5_ nanowires in the voltage ranges of 2.0–4.0 V and 1.75–4.0 V at 30 mA g^−1^. d) TEM image of V_2_O_5_ hollow microflowers. e) Cycling performance and coulombic efficiency of V_2_O_5_ hollow microflowers in the voltage ranges of 2.0–4.0 V and 2.5–4.0 V at 300 mA g^−1^. f) SEM image of V_2_O_5_ hierarchical microspheres. g) Cycling performance of V_2_O_5_ microspheres at 5 C. a) Reproduced with permission.^215^ Copyright 2016, Elsevier. b,c) Reproduced with permission.^220^ Copyright 2010, American Chemical Society. d,e) Reproduced with permission.^47^ Copyright 2013, Royal Society of Chemistry. f,g) Reproduced with permission.^223^ Copyright 2015, Elsevier.

Conventional cathode materials in LIBs have low specific capacities, which usually mismatch the specific capacity of anode materials, leading to low energy density in the practical application of full cells.^211^ Therefore, there is an increasing need for LIBs to explore alternative cathode materials with high specific capacity. Among the potential cathode materials, V_2_O_5_, which has high theoretical specific capacities (294 or 441 mA h g^−1^ based on insertion of two or three Li per formula unit, respectively),^212^, ^213^ has attracted significant attention in recent years because of its abundance, low cost, and ease of fabrication.^214^, ^215^, ^216^, ^217^ As illustrated in **Figure** **15**a, V_2_O_5_ has a layered structure. Li^+^ insertion and extraction from V_2_O_5_ framework can be expressed as: V_2_O_5_ + *x*Li^+^ + *x*e^−^ ↔ Li*_x_*V_2_O_5_.^218^, ^219^ Given the mechanism of V_2_O_5_, the architectural integrity can remain without the collapse in electrochemical cycling, resulting in long‐term stability. Therefore, V_2_O_5_ shows great potential as a desirable cathode material in LIBs.

To further enhance the electrochemical performances of V_2_O_5_, various hierarchical V_2_O_5_ nanomaterials have been studied. Mai et al. synthesized ultralong hierarchical V_2_O_5_ nanowires composed of tiny‐sized nanorods (Figure 15b) through electrospinning method.^220^ This hierarchical V_2_O_5_ exhibited higher capacity because the attachment of nanorods in ultralong nanowires reduced the self‐aggregation of nanobuilding blocks to keep the effective contact areas between the active materials and electrolyte. Electrospinning method has been extensively utilized in manufacturing ultralong hierarchical nanowires with controllable diameters, lengths, and complex architectures. The second and 50th discharge capacities of hierarchical V_2_O_5_ nanowire cathodes could attain up to 315 and 201 mA h g^−1^ when the LIBs cycled between 1.75 and 4.0 V. When cycled between 2.0 and 4.0 V, the second and 50th discharge capacities of nanowire cathodes were 270 and 187 mA h g^−1^, respectively (Figure 15c). Lou and co‐workers prepared 3D hierarchical V_2_O_5_ microflowers nanostructure (Figure 15d) through facile solvothermal strategy.^47^ When the voltage window was between 2.0 and 4.0 V, the material could retain a reversible capacity of 211 mA h g^−1^ after 100 cycles, leading to only 0.27% of the capacity fading rate per cycle (Figure 15e). A narrower voltage window scope of 2.5–4.0 V yielded a specific capacity of 140 mA h g^−1^ when cycled at 300 mA g^−1^ (Figure 15e). Moreover, the results indicated a lower capacity fading rate of 0.15% per cycle, leading to better cycling performance than the former voltage window. Zhang and co‐workers also reported a V_2_O_5_ hierarchical structure, which could achieve a capacity fading rate of 0.08% per cycle.^221^ Li et al. prepared a hierarchical flower‐like microsphere V_2_O_5_ nanostructure via solvothermal method.^222^ An ultralow capacity fading rate of 0.04% per cycle could be obtained within 500 cycles when the voltage window was controlled at a narrow range of 2.0–3.0 V during electrochemical test. Yang and co‐workers also synthesized hierarchically structured V_2_O_5_ flower‐like microspheres (Figure 15f) through a template‐free process followed by annealing.^223^ When applied in electrochemical performance testing, V_2_O_5_ microspheres delivered a high capacity of 275 mA h g^−1^ at 1 C. Furthermore, the product could maintain a high capacity of 200 mA h g^−1^ even after 500 cycles at 5 C (Figure 15g). Recently, Lou and co‐workers also developed an additive‐free solvothermal method to fabricate a hierarchical nanostructure of 3D porous V_2_O_5_ microspheres.^217^ This hierarchical V_2_O_5_ material exhibited a stable capacity of 130 mA h g^−1^ after 100 cycles at 0.5 C owing to their unique structure and delivered a capacity of 105 mA h g^−1^ even at an ultrahigh rate of 30 C, leading to a remarkable rate capability.

Hierarchical polyhedral structures of V_2_O_5_ have also been prepared as cathodes in LIBs. Mai and co‐workers prepared 3D porous hierarchical V_2_O_5_ octahedrons through facile solid‐state conversion.^224^ This 3D hierarchical nanostructure was generated from heat‐treatment of the ammonium vanadium oxide precursor. When evaluated as a cathode for LIBs at 2.4–4 V, capacities of 135 and 96 mA h g^−1^ were acquired when cycled at 100 and 2000 mA g^−1^, respectively. Moreover, 96.9% of the initial capacity was retained even after 500 cycles at 2000 mA g^−1^. Recently, Zhang et al. have manufactured a V_2_O_5_/C composite with hierarchical polyhedral structure.^225^ During the preparation, the liquid vanadium precursor was combined with the dodecahedral mesoporous carbon framework derived from zeolitic imidazolate frameworks, subsequently obtaining the product after annealing treatment. The carbon frameworks did not only play a role in the conductive network, but also functioned as a solid protective layer in preventing the structure from breaking during the charge/discharge process.^226^, ^227^ Meanwhile, the encapsulated V_2_O_5_ active nanoparticles could contact fully with electrolyte. The sample could deliver a capacity of 130 mA h g^−1^ at 5 C and retained a capacity of 98 mA h g^−1^ after 800 cycles, with a capacity retention of 75.7%.

A comparison of typical hierarchically nanostructured TMOs based on intercalation/deintercalation reaction is given in **Table** **2**.

**Table 2:** Electrochemical performances of various hierarchically nanostructured TMOs based on intercalation/deintercalation reaction

Materials	Feature	Electrochemical performance				Ref.
		Current density [mA g^−1^]	Capacity (initial cycle/second cycle) [mA h g^−1^]	Cycle number	Capacity retention [mA h g^−1^]	
TiO_2_	3D anatase TiO_2_ nanocrystal microspheres	1 C	≈210/≈185	100	174	169
	Porous anatase TiO_2_ microspheres	10 C	207.4/≈180	200	142.3	185
	TiO_2_(B) HTs	5 C	≈230/≈210	400	160	197
	Mesoporous TiO_2_(B) microspheres	10 C	–	5000	149	190
	TiO_2_ (B)‐BH	5 C	≈200/≈190	1000	186	188
	Rutile TiO_2_ microspheres	0.1 C	≈350/≈250	30	192	198
	3D TiO_2_@C core–shell structure	1 C	351/≈255	200	228	205
	Ultrafine TiO_2_ NP@PG	100	≈300/≈262.5	40	≈250	206
Nb_2_O_5_	Nb_2_O_5_/HGF	10 C	–	10 000	≈125	209
	Urchin‐like Nb_2_O_5_ microspheres	5 C	123.7/≈120	500	105.5	210
V_2_O_5_	Ultralong V_2_O_5_ nanowires	30	390/≈320	50	201	220
	3D V_2_O_5_ microflowers	300	≈275/≈280	100	211	47
	Flower‐like V_2_O_5_ microspheres	5 C	≈252/≈240	500	200	223
	3D porous V_2_O_5_ octahedrons	2000	96/≈95	500	93	224
	Polyhedron structure of V_2_O_5_/C composite	5 C	130/≈128	800	98	225

## 4 Hierarchical TMOs Based on Other Reaction Mechanisms

Most of TMOs for LIBs are based on conversion reaction or intercalation/deintercalation reaction. There remain a few TMOs, such as ZnO, ZnFe_2_O_4_, and ZnCo_2_O_4_, which are based on alloying–dealloying reaction or a combination of alloying–dealloying reaction and conversion reaction.

### 4.1 Zinc Oxides

Zinc oxide has been considered as a promising TMOs for LIB's anode material because of its high theoretical capacity (978 mA h g^−1^) and environmental friendliness. It belongs to the reaction mechanism of alloying–dealloying reaction: (1) ZnO + 2Li^+^ + 2e^−^ → Zn + Li_2_O (2) Zn + *x*Li^+^ + *x*e^−^ ↔ Li*_x_*Zn (*x* ≤ 1).^35^, ^228^ Generally, the amorphous type of ZnO can exhibit admirable electrochemical performance owing to its abundant active sites, isotropic nature, and better buffer effect. Recently, Tu et al. reported a facile route to fabricate hierarchical structured amorphous ZnO quantum dots/mesoporous carbon bubble composites (ZnO QDs/MPCB).^228^ The unique composite possessed abundant active sites, hierarchical porous structure, and interconnected conductive network. When evaluated as anode material, the ZnO QDs/MPCB composite delivered a high reversible capacity of around 1000 mA h g^−1^ at 100 mA g^−1^ and a good rate capability. Additionally, the composite exhibited a superior cycling stability with about 94% capacity retention after 400 cycles at 1000 mA g^−1^. Fan et al. prepared a hierarchical yolk–shell structured ZnO@C composite via a coprecipitation method.^229^ Such hierarchical structures also possessed the merits of large specific surface area and excellent electron conductivity. Importantly, this structure provided large cavity to buffer the volume variation and maintained the integrity of the electrode material well during the electrochemical reaction. When applied in LIBs, a high reversible capacity of 1045.2 mA h g^−1^ was obtained. Furthermore, this material possessed a good cycle stability with a stable capacity over 1000 cycles at 2 A g^−1^.

### 4.2 Mixed TMOs

As a mixed TMO, ZnM_2_O_4_ (M mainly includes Fe and Co) has attracted considerable attention because of its high theoretical capacity.^230^, ^231^, ^232^, ^233^ Due to the existence of Zn element, ZnM_2_O_4_ belongs to a special reaction mechanism based on both alloying–dealloying reaction and conversion reaction.^35^ To further enhance the electrochemical performance of ZnM_2_O_4_, hierarchical structures have been fabricated. Guo et al. reported the preparation of hierarchically hollow ZnFe_2_O_4_ microspheres through the hydrothermal reaction.^234^ The hierarchically hollow structure enhanced the specific capacity and cycling stability of ZnFe_2_O_4_. The sample delivered a high specific capacity of 1200 mA h g^−1^ for the initial cycle and the specific capacity was stabilized at about 900 mA h g^−1^ at 65 mA g^−1^ in the subsequent 50 cycles. Additionally, the sample exhibited a relative high initial Coulombic efficiency of ≈78%. Hou et al. developed a facile strategy to prepare hierarchical shuttle‐shaped mesoporous ZnFe_2_O_4_ microrods.^235^ This hierarchical structure was constructed with 1D nanofiber subunits and manifested desirable mechanical properties. When utilized as anode material for LIBs, the product delivered an initial charge capacity of ≈1150 mA h g^−1^ at 100 mA g^−1^ and an initial Coulombic efficiency of ≈76%. In addition, Yu et al. prepared hierarchically 3D porous ZnCo_2_O_4_ on macroporous nickel foam via a solution‐based method.^236^ Due to the characteristics of the interconnected porous network, numerous electroactive sites and rapid ion transfer could be achieved. The hierarchical ZnCo_2_O_4_ electrode exhibited a high specific capacity of 1200 mA h g^−1^ for the 2nd cycle at 100 mA g^−1^ and a good cycling performance (about 70% retention of the 2nd cycle capacity after 500 cycles).

## 5 Conclusions and Outlooks

In this review, we make an overview on the recent developments of hierarchically nanostructured TMOs for LIBs. Various TMOs, such as iron oxides, cobalt oxides, nickel oxides, manganese oxides, titanium oxides, niobium oxides, and vanadium oxides, have been investigated as electrodes for LIBs. These TMOs are classified on the basis of two reaction mechanisms, namely, the conversion reaction and the intercalation/deintercalation reaction. Because of their inherent features, the two types of TMOs exhibit different properties when used for LIBs. TMOs based on the conversion reaction usually possess high theoretical specific capacity. For example, iron oxides and manganese oxides can deliver a theoretical specific capacity of up to ≈1000 mA h g^−1^. However, these kinds of TMO have poor structural integrity caused by the transformation between TMOs and elemental metal during the charge/discharge process. By contrast, TMOs based on the intercalation/deintercalation reaction have relatively low theoretical specific capacity, but maintain the structural integrity and ensure good cycling stability. Hierarchically nanostructured TMOs with various morphologies, such as hierarchical nanowire, nanotube, microbox, and 3D hierarchical microspheres (including 3D hierarchical microflower and hollow spheres), have been discussed systematically. The hierarchical nanostructure can deliver a superior electrochemical performance in comparison with the nonhierarchical structure. A hierarchical nanostructure not only provides more active sites for redox reaction but also shortens the transport distance of Li^+^. Moreover, the hierarchically nanostructured TMOs can address the problem of serious volume change, which is a disadvantage of TMOs based on the conversion reaction. During electrochemical cycling, the hierarchical structure can accommodate the strains caused by volume expansion.

According to the published literature, hierarchically nanostructured TMOs prepared through rational design exhibit superior performances. To further improve the electrochemical performance, we should focus on the following several aspects:

(1) The composites of TMOs with conductive materials have superior properties over single TMOs. Conductive materials, such as nanostructured carbon materials and conductive polymers, can largely enhance the conductivity of the entire material, addressing the intrinsic poor conductivity of TMOs. This strategy is efficient in improving the rate performance of the battery.
(2) Hierarchical hollow structures usually exhibit better performance than nonhollow hierarchical architectures because the hollow architecture in the former participates in buffering the strain of volume expansion during charge/discharge process. Therefore, the design of materials with hollow structures may be a good choice for high‐performance electrode.
(3) Hierarchical nanostructure consists of nanobuilding blocks. Self‐assembly is a very efficient way of designing desirable nanostructures, for this method can avoid structural collapse during the synthetic process in contrast to the template method.
(4) Binder‐free electrodes are also an important aspect in LIB research. For traditional electrodes, the synthetic process usually needs polymeric binders or other additives to adhere the active material to the current collector. Insulating and inactive binders usually decrease the capacity and rate capability because binders decrease the electronic conductivity and block the diffusion paths of Li^+^. In this regard, binder‐free electrodes directly prepared on current collectors can solve this problem because of in situ tight adhesion.
(5) For TMOs based on conversion reaction, in addition to the disadvantage of large volume change, several other disadvantages need to be focused on as well.^15^ First, these materials usually exhibit a relatively low first cycle Coulombic efficiency. Second, compared to C or Si anodes, these materials usually need a relatively high charge potential, which will obviously reduce the full cell voltage and the energy density of the full cell. Finally, these materials usually exhibit a relatively large voltage hysteresis between charge curve and discharge curve, which will result in poor voltage efficiency and energy efficiency. Therefore, to comprehensively improve the performance of conversion materials, it is necessary to further study the causes of above problems and find solutions to overcome these problems.
(6) Although the hierarchical nanostructure provides many advantages for LIBs electrode materials, this structure also suffers from two disadvantages compared to general nanostructured materials. Its loose structure results in a low volume energy density. To address this issue, we should design more rational structures for hierarchically nanostructured TMOs. Furthermore, it must be noted that, although a high surface area for hierarchically nanostructure TMOs as anode materials might enhance the kinetic performance, it would also cause an increased electrolyte decomposition and most likely an enhanced loss of active lithium. Therefore, it is necessary to find ways to suppress the side effects.

## Conflict of Interest

The authors declare no conflict of interest.
